# Within and Among Patch Variability in Patterns of Insect Herbivory Across a Fragmented Forest Landscape

**DOI:** 10.1371/journal.pone.0150843

**Published:** 2016-03-03

**Authors:** Dorothy Y. Maguire, Christopher M. Buddle, Elena M. Bennett

**Affiliations:** 1 Department of Natural Resource Sciences, McGill University, St-Anne-De-Bellevue, Quebec, Canada; 2 School of Environment, McGill University, Montréal, Quebec, Canada; Centre for Cellular and Molecular Biology, INDIA

## Abstract

Fragmentation changes the spatial patterns of landscapes in ways that can alter the flow of materials and species; however, our understanding of the consequences of this fragmentation and flow alteration for ecosystem processes and ecosystem services remains limited. As an ecological process that affects many ecosystem services and is sensitive to fragmentation, insect herbivory is a good model system for exploring the role of fragmentation, and the resulting spatial patterns of landscapes, in the provision of ecosystem services. To refine our knowledge of how changes in landscape pattern affect insect herbivory, we quantified the combined influence of among patch (patch area and patch connectivity) and within patch (location within patch; canopy, edge, interior) factors on amounts of insect herbivory in a fragmented forest landscape. We measured herbivory in 20 forest patches of differing size and connectivity in southern Quebec (Canada). Within each patch, herbivory was quantified at the interior, edge, and canopy of sugar maple trees during the spring and summer of 2011 and 2012. Results show that connectivity affects herbivory differently depending on the location within the patch (edge, interior, canopy), an effect that would have gone unnoticed if samples were pooled across locations. These results suggest considering structure at both the patch and within patch scales may help to elucidate patterns when studying the effects of fragmentation on ecosystem processes, with implications for the services they support.

## Introduction

Human encroachment on the environment through resource extraction and urban expansion have led to the fragmentation of forests, with considerable, and often negative, consequences for biodiversity [1], ecosystem processes [2] [3], and the ecosystem services they support [4] [5] [6]. The sustainable management of such fragmented landscapes will depend on understanding the spatial ecology of the ecosystem services needed over the long-term [7] [8], which in turn, will depend on developing a thorough understanding of the spatial ecology of the ecosystem processes that support their provision [9] [10]. Incorporating the spatial heterogeneity of ecosystem processes and services into landscape scale models will help improve their accuracy [7] [11] [12], informing better sustainable management of landscapes for a diversity of services.

Forest fragmentation alters the spatial properties of patches within landscapes at multiple scales. At the patch scale, fragmentation can alter factors such as the configuration of forest patches (i.e. their location within a landscape, and their relative proximity to other patches). Fragmentation can also alter the types and amounts of habitat available for organisms within patches, including the amount and quality of edge habitat, or change the amount and quality of interior space within a patch. These multi-scale effects of fragmentation can interact to affect the spatial pattern of ecosystem processes and the delivery of services these processes support in fragmented landscapes [13].

Since ecosystem processes support ecosystem services, accounting for the effects of spatial pattern at multiple scales on ecosystem processes can lead to a more accurate understanding of how services are provisioned across landscapes. For example, [14] demonstrated that considering among- patch factors such as landscape composition and configuration on biodiversity, water and nutrient cycling, and associated ecosystem processes in a fragmented landscape improved the accuracy with which they measured services in tropical rainforests. Similarly, accounting for within- patch factors such as forest composition within patches, can lead to better assessments of the diversity and composition of communities [15] [16], which support services [17]. Within- patch factors such as edge effects may also affect ecosystem processes such as carbon storage due to edge-induced tree mortality, and changes in light availability in forests, ultimately affecting services such as climate regulation [18]. Despite the message that multiple scales of spatial pattern matters for ecosystem processes and the services they support, there is little research that quantifies these effects. Our research fills this gap by assessing patterns of herbivory—an important ecosystem process—in response to both among- and within- patch factors, and their interactions, across a fragmented forest landscape.

We use insect herbivory as a model ecosystem process for understanding the effects of spatial pattern on processes in forests. Herbivory in forests affects a broad range of processes and services by influencing primary productivity [19], regulating tree species recruitment dynamics [20], modifying tree growth, survival, and reproduction [20], altering nutrient dynamics [21] [22] [23], modifying demographics and succession of forests [24], and changing forest biodiversity [25]. By affecting these processes and biodiversity, insect herbivores can affect the services we get from ecosystems [26] [27] [28]. For example, [28] argues that depending how much defoliation occurs, herbivorous insects can have either detrimental or beneficial impacts on services such as timber production, soil formation, and carbon sequestration. Aside from total herbivory, inter- and intra- annual variation in herbivory is also an important determinant of the impact it can have on ecosystems [29] [30]. Determining the rate and variation in overall levels of defoliation in a landscape is required to shed light on the impact of this process on ecosystem services.

Herbivory is sensitive to changes in the spatial properties of patches caused by fragmentation at multiple scales. Among patches, herbivory can be affected by differences in the size and connectivity of forest patches that results from fragmentation. Reductions in in patch area have been shown to negatively affect ecosystem processes like herbivory by altering abundances of herbivores and their natural enemies in remnant forest patches [31]. These effects are exacerbated when patches are isolated from each other, and separated by a matrix of inhospitable habitat [32] [33]. Reductions in area and connectivity of patches caused by fragmentation may increase amounts of insect herbivory by reducing top-down control (predation) of insect herbivores [34] [35] [36]. Fragmentation can also decrease insect herbivory via changes in abiotic factors, such as temperature and humidity, and resource limitation (i.e. “bottom-up” factors) that affect herbivores directly [37]. A recent review of the literature indicates that reductions in connectivity can influence herbivory either negatively or positively depending on the species involved and ecosystem [28]. Patch size and connectivity are therefore key determinants of the spatial distribution of insect herbivores [38] [39].

Insect herbivory is influenced by spatial patterns within patches. Factors such as the location within the patch are important since the biotic and abiotic structures of a forest vary widely depending on whether we consider the edge, interior or canopy [40] [25]. Abiotic factors such as radiation, moisture, temperature and wind tend to be harsher, and more variable at edges and canopies alike [41] [42]. Biotic factors such as plant architecture, foliage quality and quantity, and species compositions also tend to be different in edges [41] as well as in canopies [43] [44]. Amounts of herbivory at the edges are often higher than in forest interiors [30] [45] [46] [47] due to changes in trophic interactions, such as reduced control of herbivores by natural enemies at the edges. Herbivory is generally lower in canopies than understories; predation pressure [48], leaf palatability [49] [50] and exposure to abiotic factors [51] can affect the vertical distribution of herbivores, and therefore herbivory, in these locations. Despite a long history of considering edge effects in fragmentation studies, relatively few studies consider the effects of fragmentation on forest canopies. This is surprising given that, similar to edge effects, fragmentation likely affects canopies differently than the understory and edge because of the different plant structures [43], and species [52] [53] found in these areas. Therefore forest interiors, canopies and edges seem to be important spatially segregated areas within patches that may experience different pressure from herbivores that may also respond distinctively to fragmentation.

We assessed patterns of insect herbivory relative to among-patch (size and isolation of forest patches) and within-patch (location in patch i.e. canopy, edge, interior) factors in a fragmented forest landscape to develop a better understanding of interactions between multiple scales of landscape pattern on an ecosystem process. We expect both patch configuration and location within patch to be important determinants of herbivory in this system. However, the magnitude and relative importance of these effects, as well as whether or not there is an interaction between these two factors, is unknown.

## Materials and Methods

### Study Sites and Experimental Design

**Fig 1:**
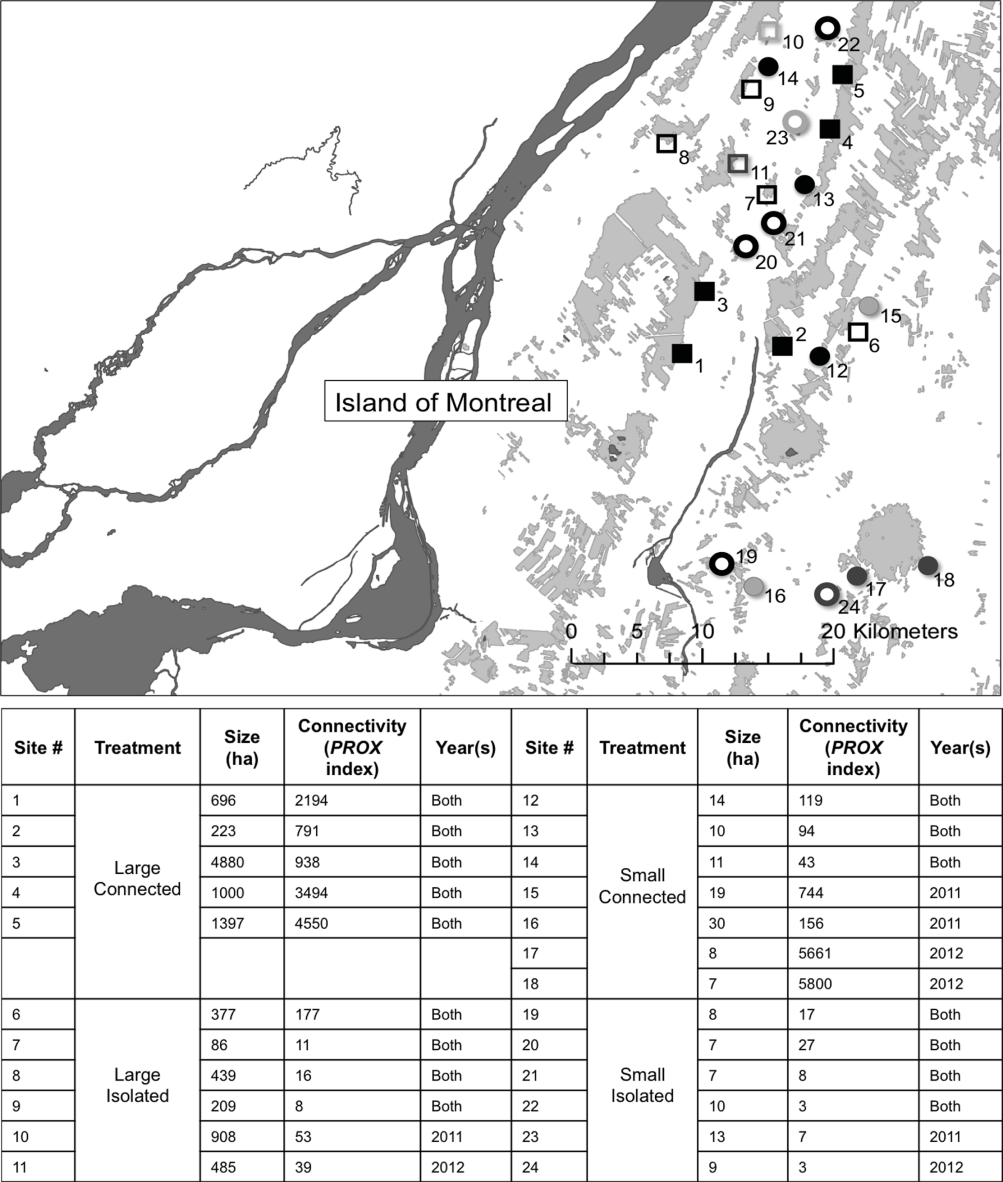
Map of Field Sites used in this study. Map of forest patches (grey shaded areas) in which herbivory data were collected in 2011 and 2012. Patches are located in southern Quebec, Canada just east of Montreal. Individual size and connectivity metrics for each patch are outlined in the figure. Size is reported in hectares, and connectivity is reported as a proximity index (*PROX*) described in [54]. Large sites are indicated by squares, while small sites are indicated by circles. Connected sites are indicated by solid shapes, isolated sites are indicated by hollow shapes. Black shapes indicate sites that were used in both years, light grey shapes are sites that were used only in 2011, and dark grey shapes are sites that were used only in 2012. Complete site characteristics and GPS coordinates are included in S2 Table.

Our study was conducted in the Montérégie of southwestern Quebec (Canada) throughout the spring and summer of 2011 and 2012. In each year, 20 forest patches were selected (Fig 1). Permission was granted by land owners prior to accessing private land. For this reason 16 patches were used in both years, and 8 were used only in one year or the other due to issues related to re-establishing contact with some land owners in the second year. The forest patches in the region are a diverse mixture of deciduous and coniferous forest located within a corn- soy agricultural matrix. We sampled only in forest patches that were temperate sugar maple-beech forests (*Acer saccarum* Marsh.*—Fagus grandifolia* Ehrh.) because this represented the dominant forest type in the landscape. Particular fragments were selected for inclusion based on area and connectivity (as measured using the FRAGSTATS proximity index (*PROX*), which takes into account the distance between the focal patch and its neighbors as well as the total area of adjacent patches within a 2 km boundary [54]). We first sorted all patches in the landscape by size. We then separated all forest patches into those we classified as small patches (less than 30 ha) and those we considered large patches (greater than 85 ha). Within these size classes, we sorted a list of candidate patches by their proximity index where isolated sites were those with the lowest *PROX* index and connected sites had the highest *PROX* index. Ultimately, we selected five patches in each of the following four types: five small isolated (7–30 ha, *PROX* values <30), five small connected (7–30 ha, *PROX* values >43), five large isolated (85–4900 ha, with *PROX* values <177), and five large connected (85–4900 ha, with *PROX* values >223). *PROX* values for isolated and connected patches are not equivalent for large and small patches because *PROX* indices were correlated with patch size.

### Sampling

We assessed herbivory on sugar maple trees in five rounds that took place from May to September, in 2011 and 2012 (Round 1 = late May- early June, Round 2 = late June- early July, Round 3 = late July- early August, Round 4 = mid- late August, Round 5 = early- mid September). We sampled herbivory on sugar maple trees in three locations: at the edge, the interior, and the canopy of forest patches. In each patch, 6 similarly aged sugar maple saplings (4–5 cm diameter and 1.6–2 m tall) were selected at both the edge and the interior of forest patches, and 2 mature sugar maple canopy trees (20–40 cm DBH) were selected at the interior of forest patches. In order to separate effects of vertical stratification from tree ontogeny as best as possible, leaves were collected at a height of approximately16–18 m in canopy trees, compared to a max height of just 2 m in understory trees.

From saplings at the edge and interior, we randomly selected 10 leaves per tree sapling (representing >12% of total leaves on each sapling [55]). From canopy trees, we randomly selected 30 leaves (10 from each of three randomly selected branches, about 15% of total leaves per branch) [56] [50]. Although understory sugar maple leaves typically flush 5d earlier than canopy leaves [57], leaves in both understory and canopy trees were fully flushed when we started and ended sampling to minimize bias associated with differences in leaf ontogeny. We categorized leaves based on the percentage of missing/damaged leaf area caused by leaf chewing and sucking arthropod herbivores (category 0 = 0%, 1 = 1–5%, 2 = 6–15%, 3 = 16–25%, 4 = 26–50%, 5 = 51–75%, 6 = 76–100%). To do this, we constructed a visual guide using leaves that were analyzed for percentage damage with imaging software (ImageJ). After leaves were placed into damage categories using the guide, we calculated a herbivory index (H) for each sample to quantify the total amount of leaf area eaten by insects: *H =* ∑ *n*_*i*_*(C*_*i*_*)/N*, where *i* is the damage category, *n*_*i*_ is the number of leaves in the *i*th damage category, *C*_*i*_ is the midpoint of each category (i.e. C_0_ = 0%, C_1_ = 3%, C_2_ = 10.5%, C_3_ = 20.5%, C_4_ = 38%, C_5_ = 63%, C_6_ = 88%), and *N* is the total number of leaves sampled on the tree [58] [59] [60]. The index formula was modified to include more damage categories than in the original publication to detect finer scale differences in herbivory levels. Raw herbivory data are included in S3 Table.

### Statistical Analyses

We tested the effects of time of season (sampling rounds 1–5), patch connectivity (isolated versus connected patches), area (large versus small patches), location within forest patch (interior, edge, canopy), and all possible interactions on amounts of herbivory (measured as a herbivory index). Years were treated separately in all analyses since forest patches and trees were not necessarily consistent between years. All data were analyzed in R [61] using linear mixed effects models in the package nlme [62], since our design contains both random and fixed effects. We started with a fully fitted model that included all main effects (size, connectivity, location within patches, sampling round), all possible interactions, and random effects (individuals or “trees” nested within forest patches). This random effects structure allows us to account for patch effects, and repeated measures on trees. Herbivory data were log transformed prior to analysis in order to meet assumptions of homogeneity and normality. In addition, the spread of our data varied among locations, and over time, so we accounted for these unequal variances by specifying the variance structure in our model. Model fitting and checking procedures were carried out as recommended in [63] by fitting the full model, and finding the optimal random and fixed structure. Finally, the fit of these models to our data was evaluated using plots of fitted values vs the standardized residuals. Statistical significance of factors in our model was ascertained using likelihood ratio tests as is recommended by [63]. In order to assess statistical differences among levels of treatments in our model, for example assess edge effects (edge understory versus interior understory), and effects of vertical stratification (canopy versus interior understory) we performed Tukey’s HSD multiple comparisons tests of all pairwise combinations of factor levels when our results indicated significant treatment effects. Since coefficients better illustrate significance of factors in mixed models, coefficients +/- 95% confidence intervals extracted from the final mixed models are reported in S1 Fig.

## Results

**Fig 2:**
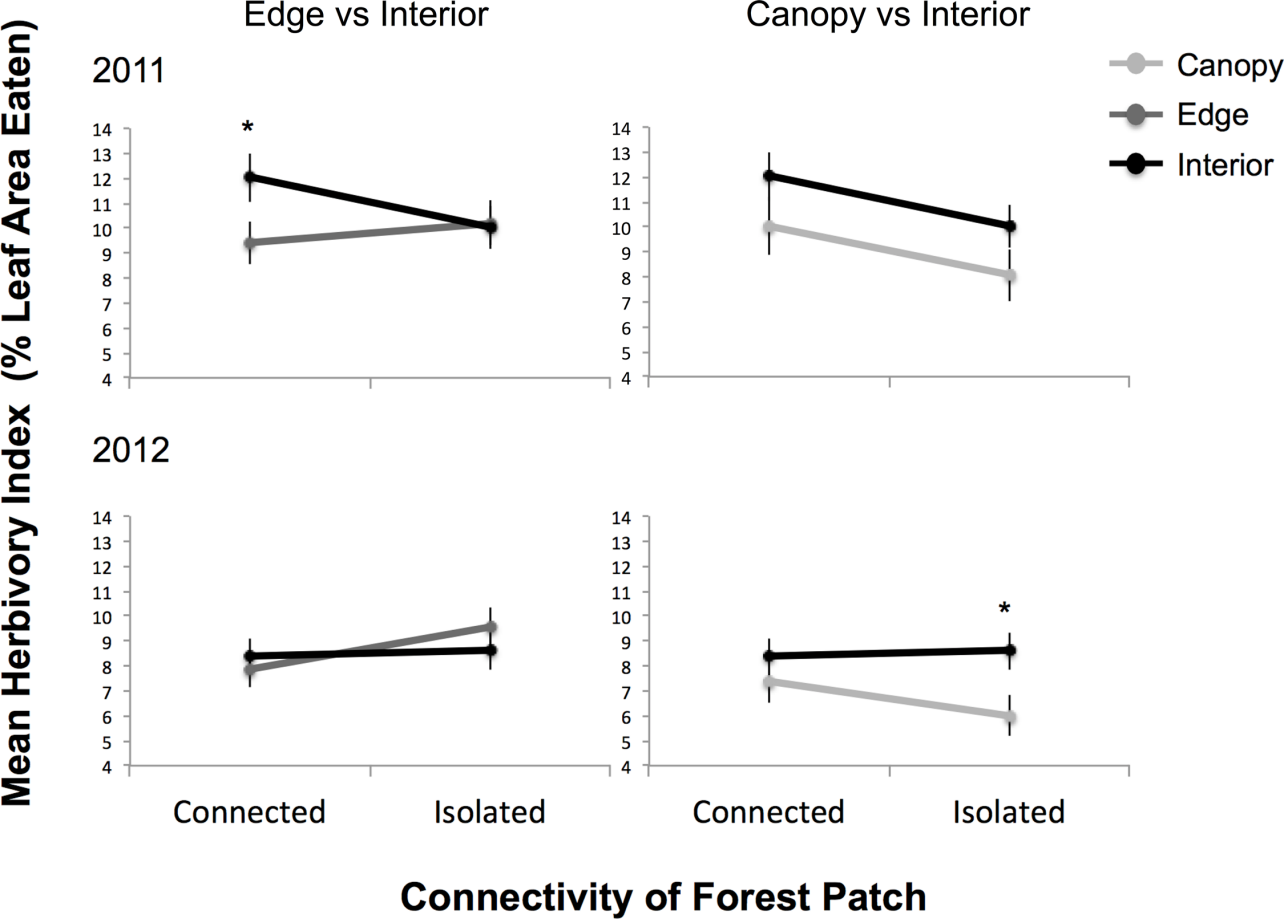
Herbivory by patch connectivity and location within patch. Mean herbivory index (+/-SE) for patches with different connectivities (connected vs. isolated), and for different locations within patches (edge vs. interior understory, and canopy vs interior understory). Means and standard errors are back-transformed predicted values from the mixed effects model with trees nested within patches as random effects. Significant results of multiple comparisons tests are indicated by a star (*), with Interior Connected > Edge Connected in 2011 (p<0.05), and Canopy Isolated < Edge Isolated (p<0.0001) and < Interior Isolated (p<0.05) in 2012.

The amount of herbivory in our study depended on the interaction between both among- and within- patch factors, as was evidenced by a significant interaction term between forest patch connectivity (isolated versus connected) and location within forest patch (interior versus edge versus canopy). This overall interaction effect was marginally insignificant in 2011 (p = 0.0659), but more pronounced in 2012 (p = 0.0266) (Table 1). When we examined the edge effects (edge understory versus interior understory), and effects of vertical stratification (canopy versus interior understory) in more detail using multiple comparison tests of these results, we showed that, compared to forest interiors, edges had significantly less herbivory in connected patches, but only in 2011 (Fig 2)(Table 1). Compared to the forest understory, canopies had significantly less herbivory, but only in isolated patches in 2012 (Fig 2)(Table 1). These results indicate that in a given year, connectivity is influencing herbivory differently among locations within patches.

**Table 1:** Results of mixed effects models testing the effects of sampling period, landscape characteristics and locations within forest patches on herbivory.

Year	Factor	DF	F-value	p-value	Comparisons
2011	Patch Area	1, 16	0.23	0.6381	
	Patch Connectivity	1, 16	0.56	0.4641	
	Location Within Patch	2, 254	4.27	0.0150	
	Time of Season	4, 875	15.37	**< .0001**	R2<R1*** R3>R2*** R3 = R1 R4>R2*** R4 = R1, R3 R5>R2,R4*** R5>R1 * R5 = R3
	Patch Area x Patch Connectivity	1, 16	0.07	0.8001	
	Patch Area x Location Within Patch	2, 254	0.90	0.4085	
	Patch Connectivity x Location Within Patch	2, 254	2.75	**0.0659**	**Connected-Interior> Connected-Edge** *
2012	Patch Area	1, 16	0.79	0.3882	
	Patch Connectivity	1, 16	0.36	0.5587	
	Location Within Patch	2, 254	6.10	0.0026	
	Time of Season	4, 1116	59.60	**< .0001**	**R2>R1** ** R3>R2, R1 *** R4>R2, R1 *** R4 = R3 **R5>R3, R2, R1** *** R5 = R4
	Patch Area x Patch Connectivity	1, 16	0.25	0.6234	
	Patch Area x Location Within Patch	2, 254	1.03	0.3585	
	Patch Connectivity x Location Within Patch	2, 254	3.68	**0.0266**	**Isolated-Edge> Isolated-Canopy** *** Isolated-Interior> Isolated-Canopy *

Models tested the fixed effects of sampling period (sampling rounds 1–5), patch size (large vs small), patch connectivity (connected vs. isolated) and locations within patches (canopy vs edge vs interior) on herbivory in 2011 and 2012 while considering the random effects of tree and forest patch (not shown). In both years, herbivory was measured on 12 understory sugar maple trees (6 in the interior, and 6 at the edge) and 2 canopy sugar maple trees in 20 forest patches throughout southern Quebec. Comparisons between sampling rounds and the interactions between patch connectivity and location within forest patches are presented

(***p<0.0001

**p<0.01

*p<0.05).

**Fig 3:**
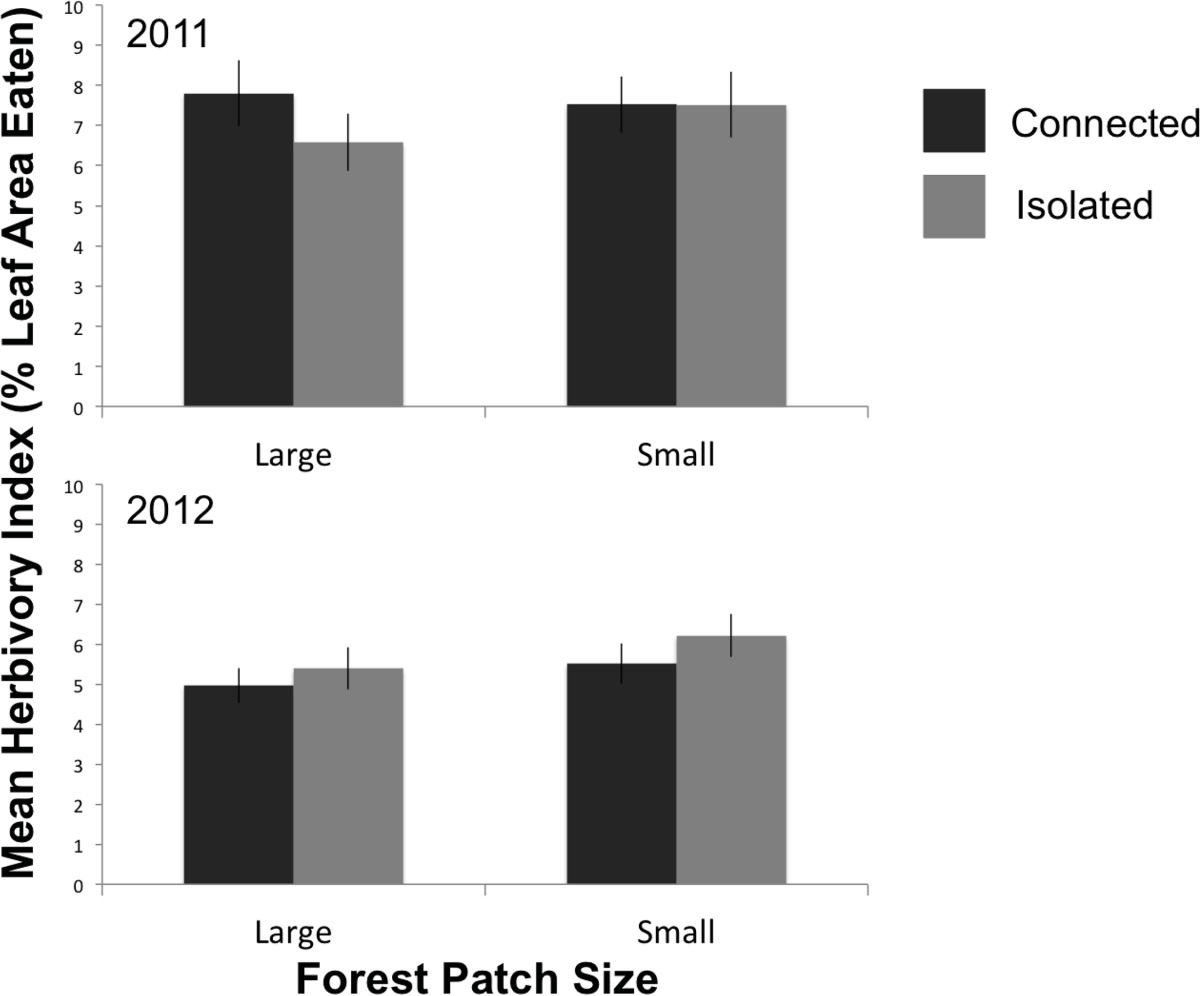
Herbivory by forest patch landscape characteristics. Mean herbivory index (+/- SE) for different forest patch sizes (large vs. small) and connectivity (isolated vs. connected) for both 2011 and 2012. There were no significant differences among treatments when data for the different locations were pooled.

Amounts of herbivory did not depend on landscape level factors when data were pooled across locations (i.e. canopy, edge, interior) within forest patches. There was no effect of forest patch size on amounts of insect herbivory in either 2011 or 2012 (Fig 3)(Table 1). There was also no effect of forest patch connectivity (isolated forest patches versus connected) on herbivory in either year (Fig 3)(Table 1).

**Fig 4:**
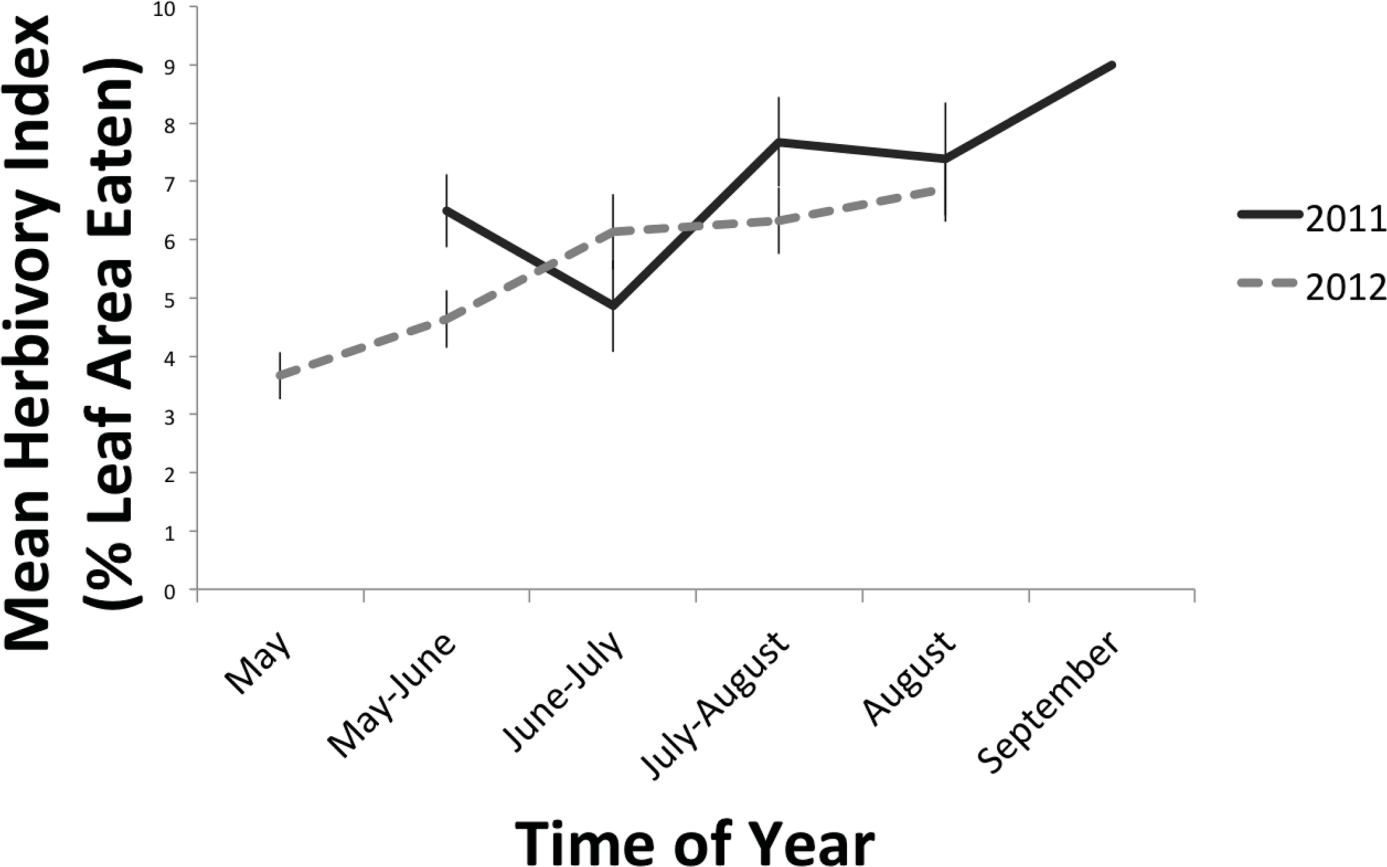
Herbivory over the course of the sampling season. Mean herbivory index (+/-SE) for each of 5 sampling rounds performed throughout the spring and summer in 2011 and 2012. In both years, herbivory was measured on 12 understory trees and 2 canopy trees in 20 forest patches throughout southern Quebec. There was a significant increase in herbivory with time of season in both 2011 and 2012 (p<0.0001).

Herbivory increased over time within the season. We found a significant effect of sampling round on the amount of insect herbivory in both 2011 (p<0.0001) and 2012 (p<0.0001)(Table 1). Levels of herbivory generally increased steadily over time with the highest levels appearing towards the end of the sampling season (Fig 4). The amount of herbivory measured peaked at 10% in 2011, and 7% in 2012. For a complete list of herbivore families collected, refer to [64] and S1 Table.

## Discussion

The objective of this research was to assess patterns of insect herbivory relative to among- and within- patch factors in a fragmented forest landscape. We observed that among- patch factors alone did not explain all the patterns of herbivory in forest patches of southwestern Quebec, even though herbivory levels have been shown to vary in response to fragmentation and changes in landscape structure (e.g. [65] [38] [35] [36] [60]). Instead, we found that the interaction of among- and within- patch factors played a key role in determining spatial patterns of herbivory across this forested landscape.

Herbivory levels in forest edges responded differently to patch connectivity than other locations within patches. Edges had more herbivory in isolated than connected patches in one year. In our study, nearby forest patches may be providing a physical buffer from disturbances associated with fragment edges, reducing the effect of edges on herbivory in well connected patches. Additionally, some species may be more likely to traverse the matrix to other patches if the patches are close to one another [66]. If the edges of more structurally connected forest patches are thus exposed to different insect communities, then it makes sense that we found different amounts of herbivory at the edges of those connected patches. Additionally, the insect communities at edges may also be different in isolated and connected patches due to differences in plant community composition, which we did not quantify in this study.

Herbivory levels in forest canopies responded differently to patch connectivity than other understory locations within patches. Canopies of isolated patches had less herbivory than those of connected patches in one year. Among patch factors such as connectivity may affect canopies differently than other within patch locations because canopies are structurally different (i.e., plant physical architecture, foliage quality and quantity) and species [25] than other locations within patches. Forest canopies also have different exposures to abiotic factors such as temperature, wind and radiation [42], which can be affected by connectivity. For example, [67] found that the extent of the effect of fragmentation on water loss in tree canopies in the Amazon was dependent on the broader landscape context. Highly fragmented (less connected) landscapes suffered more extensive water loss to canopies than less fragmented (more connected) landscapes [67], and connected forests provided a physical buffer from the elements, which helped to prevent desiccation in tree canopies caused by radiation and wind. The reduced levels of herbivory we observed in the canopies of isolated patches (relative to connected patches) may be due to such differences in abiotic conditions experienced by canopies within different landscape contexts. Since we observed no significant interaction between sampling round and location, we believe that the differences in herbivory between the canopy and understory were not due to any bias associated with slight variation in leaf ontogeny between the understory and canopy trees. It is important to note that other factors such as tree ontogeny may also explain our results since we compared leaves of understory saplings to those of mature trees. Foliar chemistry, for example, may be different between mature trees and saplings and could have had an effect on herbivory [68]. While we believe that by collecting leaves from such great differences in height, vertical stratification is likely playing a stronger role than tree ontogeny, future studies would benefit from including leaves from the lower canopy in order to parse out these effects. Taken together these results suggest that, analogous to edges, processes in canopies may respond in distinctive ways to fragmentation.

We found no significant effect of among patch factors on herbivory when data were pooled among locations. This is consistent with recent studies of herbivory in forest fragments that found overall levels of herbivory were similar between fragmented and continuous forests [69] [34] [70]. Herbivory is a complex process that may not be dependent on simple landscape metrics or resource availability, but may depend instead on interactions among a range of drivers operating across scales, including the dispersal ability of species involved, the scale at which herbivores and their natural enemies respond to fragmentation, and species-specific responses to human disturbances. Further, the different effects of fragmentation on herbivory may ultimately be somewhat hidden due to variability that emerges as a result of interactions of fragmentation with finer scale, within patch factors.

Levels of herbivory observed in this landscape are consistent with other studies that have examined endemic levels of herbivory in temperate forest ecosystems [50]. We found that herbivory increased over the course of the season, which makes sense since sugar maple leaves flush once in the spring [71], are fed upon by herbivores, and continue to accrue damage until the fall. The annual variation in levels of herbivory in response to connectivity that we found is not surprising since factors such as temperature and rainfall have been shown to influence yearly variation in herbivory [30]. We did observe an increase in herbivory over time which suggests that at least when leaves are fully flushed, there is little fluctuation in the rate of herbivory with leaf ontogeny. While the differences in amounts of herbivory we observed between locations in forest patches were marginal (fluctuations of 1–3%), further research is needed to understand the biological significance of such variation in levels of this process, and whether such changes affect biomass accumulation and productivity which are likely to have impact on other ecosystem processes and services [72]. It would also be interesting to assess whether the effects of connectivity on herbivory will be exacerbated or dampened during times of insect outbreaks. This information is important since levels of herbivory determine its effects on ecosystem services [28] [26]. For example recent research has demonstrated the importance of incorporating variation in levels of herbivory into landscape level models of ecosystem services such as carbon sequestration. They show that herbivory will increase in response to elevated CO_2_ and could affect the ability of forests to serve as carbon sinks in scenarios of climate change [73].

## Conclusions

As human fragmentation of natural areas continues to escalate, sustainable management of fragmented landscapes will depend on a deep understanding of the spatial ecology of the processes and services we wish to conserve [7] [8]. We measured spatial patterns of herbivory across a fragmented forest landscape in response to a combination of among and within patch factors. We found that within patch location is important for understanding the effects of landscape-level fragmentation on herbivory and that important effects of spatial pattern can be missed when pooling data across within patch locations, or by only focusing on one location. This, in turn, has implications for accurate modeling of the effects of fragmentation on herbivory and other ecosystem processes, as well as understanding of the ecosystem services they support. Our results confirm the importance of incorporating multiple scales of spatial heterogeneity to develop accurate spatially explicit models of ecosystem processes and services, thereby illustrating some of the yet undocumented links between landscape pattern and ecosystem process.

## Supporting Information

S1 Fig Additional results of mixed models, 2011 and 2012. Estimated coefficients +/- 95% confidence intervals. Models tested the fixed effects of time of season (RND = 1–5), patch area (AREA = large vs small), patch connectivity (CON = connected vs. isolated) and locations within patches (LOC = canopy vs edge vs interior) on herbivory in both years seperately while considering the random effects of tree and forest patch (not shown). In both years, herbivory was measured on 12 understory sugar maple trees (6 in the interior, and 6 at the edge) and 2 canopy sugar maple trees in 20 forest patches throughout southern Quebec. Coefficients are significantly different from the baseline value when CI’s do not overlap each other or the zero line.
(EPS)

S1 Table List of all herbivore families collected. Arthropods were collected from the foliage of sugar maple trees in southern Quebec at the edge (EDG), interior (INT) and canopy (CAN) of sugar maple-beech dominated forest patches in 2011 and 2012. Forest patches were large connected (LC), large isolated (LI), small connected (SC), or small isolated (SI). See [64] Maguire et al. (2015b) for detailed methods as to how arthropods were collected.
(DOCX)

S2 Table Characteristics of field sites used in this study. Detailed breakdown of the forest patches used as field sites in this study. Provided are precise GPS coordinates of patches, the years they were used, patch size (in hectares) and patch connectivity (measured as a proximity index [54]).
(XLSX)

S3 Table Raw herbivory data. Raw data collected on herbivory used for analyses in this study.
(XLSX)
