# Supplementary material for: Within and Among Patch Variability in Patterns of Insect Herbivory Across a Fragmented Forest Landscape
Source: PLoS One. 2016 Mar 3;11(3):e0150843. doi: 10.1371/journal.pone.0150843 (PMC4777537; doi:10.1371/journal.pone.0150843)
Supplement: S1 Table — Arthropods were collected from the foliage of sugar maple trees in southern Quebec at the edge (EDG), interior (INT) and canopy (CAN) of sugar maple-beech dominated forest patches in 2011 and 2012. Forest patches were large connected (LC), large isolated (LI), small connected (SC), or small isolated (SI). See [64] Maguire et al. (2015b) for detailed methods as to how arthropods were collected. (DOCX) [file pone.0150843.s002.docx]

|  | | | **Habitat Type** | | | | | | |
| --- | --- | --- | --- | --- | --- | --- | --- | --- | --- |
| **Group** | | **Family** | **Patch Type** | | | | **Location in Patch** | | |
|  |  |  | **LC** | **LI** | **SC** | **SI** | **CAN** | **EDG** | **INT** |
| Acari | Tetranychidae | | X | X | X | X | X | X | X |
|  | Eriophyidae | |  | X | X |  | X |  |  |
| Hemiptera | Aphidae | | X | X | X | X | X | X | X |
|  | Acanthosomatidae | |  | X |  |  | X |  |  |
|  | Pentatomidae | | X | X | X | X | X | X | X |
|  | Psyllidae | |  | X |  | X | X |  | X |
|  | Drepanosiphidae | | X | X | X | X | X | X | X |
|  | Cicadellidae | | X | X | X | X | X | X | X |
|  | Tingidae | | X | X | X |  | X | X |  |
|  | Miridae | | X | X | X | X | X | X | X |
|  | Coreidae | | X | X | X |  |  | X |  |
|  | Cicadidae | | X | X |  |  | X | X |  |
|  | Cercopidae | | X | X | X | X | X | X | X |
|  | Membracidae | | X |  |  | X |  | X |  |
|  | Cixiidae | | X | X |  |  |  | X |  |
|  | Flattidae | | X | X | X |  |  | X | X |
|  | Derbidae | |  |  | X |  |  | X |  |
|  | Pemphigidae | | X | X | X | X | X |  | X |
| Coleoptera | Anobiidae | | X | X | X | X | X | X | X |
|  | Chrysomelidae | | X | X | X | X |  | X | X |
|  | Curculionidae | | X | X | X | X | X | X | X |
|  | Elateridae | | X | X | X | X | X | X | X |
|  | Tenebrionidae | | X | X | X | X | X | X | X |
|  | Scarabidae | |  |  |  | X | X |  |  |
|  | Aderidae | |  |  | X |  |  | X |  |
|  | Buprestidae | | X |  |  | X |  | X | X |
|  | Cantharidae | |  | X |  |  |  |  | X |
|  | Cerambycidae | | X |  |  |  |  | X |  |
|  | Mordellidae | |  | X |  |  |  | X | X |
|  | Lampyridae | |  | X |  | X | X |  |  |
| Hymenoptera | Tenthredinidae | |  | X | X |  |  | X |  |
| Lepidoptera | Noctuidae | | X | X | X | X | X | X | X |
|  | Lymantriidae | | X |  | X | X | X | X | X |
|  | Geometridae | | X | X | X | X | X | X | X |
|  | Lasiocampidae | |  | X |  |  | X |  | X |
|  | Pantheidae | |  |  | X |  | X |  |  |
|  | Notodontidae | | X | X | X | X | X | X |  |
|  | Limacodidae | | X |  | X |  | X | X |  |
|  | Psychididae | |  | X |  |  |  | X | X |
|  | Arctiidae | | X | X | X |  | X | X | X |
|  | Micro-lepidoptera | | X | X | X | X | X | X | X |

**S1 Table.** **List of all herbivore families collected.** Arthropods were collected from the foliage of sugar maple trees in southern Quebec at the edge (EDG), interior (INT) and canopy (CAN) of sugar maple-beech dominated forest patches in 2011 and 2012. Forest patches were large connected (LC), large isolated (LI), small connected (SC), or small isolated (SI). See [64] Maguire et al. (2015b) for detailed methods as to how arthropods were collected.
